# Assessing the Safety of Direct-Acting Antiviral Agents for Hepatitis C

**DOI:** 10.1001/jamanetworkopen.2019.4765

**Published:** 2019-06-07

**Authors:** Elizabeth A. McGlynn, John L. Adams, Jason Kramer, Amandeep K. Sahota, Michael J. Silverberg, Elizabeth Shenkman, David R. Nelson

**Affiliations:** 1 Kaiser Permanente Research, Kaiser Permanente, Pasadena, California; 2 Kaiser Permanente Center for Effectiveness and Safety Research, Kaiser Permanente, Pasadena, California; 3 Department of Internal Medicine, Transplant Hepatology, Southern California Permanente Medical Group, Los Angeles; 4 Division of Research, Kaiser Permanente Northern California, Oakland; 5 Department of Health Outcomes & Biomedical Informatics, University of Florida College of Medicine, Gainesville; 6 Department of Medicine, University of Florida College of Medicine, Gainesville

## Abstract

**Importance:**

Recent reports based on the US Food and Drug Administration’s voluntary Adverse Events Reporting System raised questions about the safety of direct-acting antivirals (DAAs) for treatment of the hepatitis C virus (HCV).

**Objective:**

To assess the rates of adverse events in patients with HCV infection exposed to DAAs compared with those not exposed.

**Design, Setting, and Participants:**

A retrospective cohort study calculated unadjusted adverse event rates for exposed vs unexposed time, using claims and clinical data from 3 health systems between January 1, 2012, and December 31, 2017. Of 82 419 eligible adults, a total of 33 808 who met eligibility criteria (age, 18-88 years; HCV quantitative result or genotype from 2012 or later; continuously enrolled; naive to DAA treatment at baseline) were included. Marginal structural modeling methods were used to adjust time-to-event analyses for characteristics that are associated with both outcomes and probability of treatment.

**Interventions or Exposures:**

Exposure to DAAs compared with no DAA exposure.

**Main Outcomes and Measures:**

Death, multiple organ failure, liver cancer, hepatic decompensation, acute-on-chronic liver event, acute myocardial infarction, ischemic or hemorrhagic stroke, arrhythmia, acute kidney failure, nonliver cancer, hepatitis B reactivation, hospitalizations, and emergency department visits.

**Results:**

Of the 33 808 patients who met all inclusion criteria, 20 899 (61.8%) were men; mean (SD) age was 57.2 (10.6) years. In unadjusted analyses, DAA exposure was associated with significantly lower rates of death (10.7 vs 33.7 events per 1000 person-years; rate ratio [RR], 0.32, 95% CI, 0.25-0.40). Seven other unadjusted adverse clinical events ratios were below 70% and statistically significant favoring the DAA group: multiple organ failure (RR, 0.56; 95% CI, 0.44-0.72), liver cancer (RR, 0.62; 95% CI, 0.48-0.80), hepatic decompensation (RR, 0.62; 95% CI, 0.52-0.73), acute-on-chronic liver event (RR, 0.68; 95% CI, 0.56-0.84), acute myocardial infarction (RR, 0.64; 95% CI, 0.42-0.97), ischemic stroke (RR, 0.63; 95% CI, 0.42-0.95), and hemorrhagic stroke (RR, 0.47; 95% CI, 0.25-0.89); none favored the non-DAA group. In the marginal structural modeling–adjusted analysis, DAA exposure was associated with statistically significant lower odds of adverse events than non-DAA exposure for death (adjusted odds ratio [aOR], 0.42; 95% CI, 0.30-0.59), multiple organ failure (aOR, 0.67; 95% CI, 0.49-0.90), hepatic decompensation (aOR, 0.61; 95% CI, 0.49-0.76), acute-on-chronic liver event (aOR, 0.71; 95% CI, 0.56-0.91), and arrhythmia (aOR, 0.47; 95% CI, 0.25-0.88).

**Conclusions and Relevance:**

Direct-acting antiviral exposure may not be associated with higher rates of any serious adverse events, including those related to liver, kidney, and cardiovascular systems. Safety concerns based on previous reports did not appear to be supported in this study with more comprehensive data and rigorous statistical methods.

## Key Points

**Question:**

Are patients with hepatitis C who receive direct-acting antivirals at increased risk for adverse events compared with those who do not receive these agents?

**Findings:**

In this cohort study of 33 808 patients in 3 health systems, direct-acting antiviral exposure was associated with lower odds of experiencing the following adverse events: death, multiple organ failure, hepatic decompensation, acute-on-chronic liver event, and arrhythmia.

**Meaning:**

Concerns about safety risks based on analyses of the US Food and Drug Administration’s Adverse Events Reporting System did not appear to be confirmed, suggesting that dispensed direct-acting antivirals may be safe for patients with hepatitis C.

## Teaser

This cohort study evaluates adverse events in patients with hepatitis C virus who are receiving direct-acting antiviral medications.

## Introduction

Approximately 2.4 million US individuals are currently infected with the hepatitis C virus (HCV)^1^ and 28% of those with chronic HCV have cirrhosis.^2^ Annually 1% to 4% of individuals with cirrhosis will develop liver cancer.^3^ Antiviral treatments for HCV previously required a combination of agents taken over 24 to 48 weeks, were associated with significant adverse effects, and were effective in 54% to 63% of patients who completed treatment.^4,5,6^ Lower rates of effectiveness were reported in urban patients in minority racial/ethnic groups.^7^ Thus, the advent of newer direct-acting antivirals (DAAs) that could be administered over 8 to 12 weeks with few significant adverse effects^8,9^ and sustained virologic response of 93% to 99% across different target populations and treatment regimens^10,11,12^ was considered a substantial breakthrough in treating HCV.^13^

Enthusiasm for DAAs was somewhat tempered by a boxed warning issued in October 2016 by the US Food and Drug Administration (FDA) about the potential for reactivation of the hepatitis B virus (HBV) among coinfected individuals.^14^ This finding prompted the Institute for Safe Medication Practices to analyze the FDA’s Adverse Events Reporting System. They reported 500 cases of liver failure and 1000 cases of severe liver injury among patients taking DAAs over 12 months ending June 30, 2016.^15^ The authors acknowledged some of the limitations of using the Adverse Events Reporting System data including the voluntary nature of the reporting, lack of detailed patient medical history data, and the possibility of some misclassification because the adverse events of interest are also significant complications of the disease. However, the authors recommended further investigation because of the large number of cases and that approximately 90% of reports were from health professionals.

Postmarketing surveillance is frequently required by the FDA as a condition of approval, particularly among new drugs that have progressed quickly through the approval process. To enable more rapid surveillance, in 2008, the FDA pioneered the use of real-world evidence through the Sentinel Initiative,^16^ which complements the Adverse Events Reporting System by enabling more in-depth investigation of safety concerns that emerge through voluntary reporting. The Sentinel Initiative uses a common data model that harmonizes data on nearly 200 million people receiving care in about 18 health systems. More recently, the Patient-Centered Outcomes Research Institute created the National Patient-Centered Clinical Research Network (PCORnet) to advance the use of real-world evidence for patient-centered studies including both comparative effectiveness and safety research.^17^ PCORnet is a large, highly representative, national network of networks with a Sentinel Initiative–based common data model. We used rigorous statistical methods on the rich longitudinal data from 3 PCORnet systems to examine whether patients with HCV who were dispensed newer DAAs experienced higher rates of adverse events than patients with HCV who were not dispensed DAAs.

## Methods

### Study Design

We conducted a retrospective cohort study using administrative, longitudinal electronic health record and other data collected during the normal course of patient care from January 1, 2012, to December 31, 2017, in 3 health systems. All participants contribute person-time in the untreated (no DAA) group until they fill a prescription for a DAA at which time they contribute person-time to the DAA group until they experience the adverse event of interest or are censored (leave the health system, end of the observation or study period). The study was approved by the Kaiser Permanente Southern California Institutional Review Board and the OneFlorida Institutional Review Board; the Kaiser Permanente Northern California Institutional Review Board ceded to the Southern California Institutional Review Board. The need for patient informed consent was waived by all institutional review boards. Each site conducted its own analyses so the identified data did not leave the study site. The study followed the Strengthening the Reporting of Observational Studies in Epidemiology (STROBE) reporting guideline for reporting observational studies.^18^

### Study Data and Setting

The study was conducted in 3 health systems: Kaiser Permanente Southern California, which serves about 4.5 million members at 15 hospital-based medical centers and 231 medical offices; Kaiser Permanente Northern California, which serves about 4.3 million members at 21 hospital-based medical centers and 247 medical offices; and OneFlorida, whose partners provide health care to more than 10 million Floridians in 22 hospitals and 1240 practice or clinic settings. The systems have complete data capture for the patients included in the study. Data sources included enrollment files, encounters across all settings, diagnoses associated with encounters, laboratory studies and results, and pharmacy dispensing. Diagnoses were coded according to the *International Classification of Diseases, 9th Revision, Clinical Modification*,^19^ and* International Classification of Diseases, Tenth Revision, Clinical Modification (ICD-10-CM)*.^20^

### Participants

**Figure 1.**
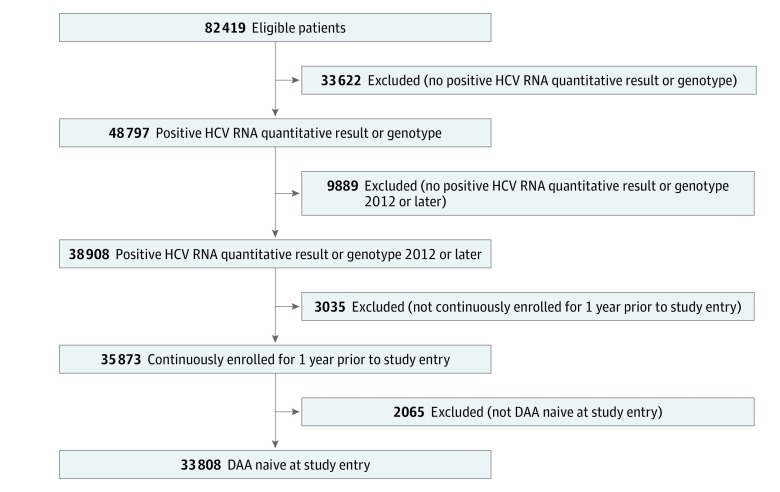
Flow of Steps to Determine Study Eligibility DAA indicates direct-acting antiviral; HCV, hepatitis C virus.

Using clinical and enrollment data from each system, we identified all adults aged 18 to 88 years who had any indication of an HCV diagnosis (genotype, quantitative or qualitative HCV viral laboratory result, HCV antibody result, *ICD* code, or medication) and who received care anytime between 2012 and 2017. We further required that patients have an HCV RNA quantitative result or genotype indicating active virus after January 1, 2012; be continuously enrolled 1 year before the index date; and be naive to DAA treatment at study entry (Figure 1).

### Exposure and Outcomes

Exposure was calculated as person-time in the non-DAA and/or DAA group. Entry to the DAA group was triggered on the date patients were dispensed their first prescription for a DAA. The outcomes of interest were serious adverse events: death, multiple organ failure, liver cancer, hepatic decompensation, acute-on-chronic liver event, acute myocardial infarction (AMI), ischemic or hemorrhagic stroke, arrhythmia, acute kidney failure, nonliver cancer, and HBV reactivation. We also examined hospitalizations and emergency department visits. Outcome and covariate definitions are provided in eTable 1 in the Supplement.

We included the outcomes of interest that are most commonly evaluated by the FDA, including liver-related events, and those recommended by 2 of us (A.K.S., D.R.N.). Outcomes were assessed from November 1, 2013, (the first month in which a DAA could have been prescribed) through December 31, 2017. Patients were followed up for up to 180 days after DAA dispensing to restrict the analysis to a time in which adverse events were most likely to be attributable to exposure to a DAA.

For AMI, ischemic stroke, hemorrhagic stroke, multiple organ failure, and arrhythmia, we required the incident appearance of *ICD* codes to be in an inpatient setting. Codes for acute kidney failure and cancers were identified from either inpatient or outpatient settings.

We constructed hepatic decompensation as a composite variable using the first occurrence of any component: variceal hemorrhage, jaundice, ascites, or hepatic encephalopathy. Because *ICD-9-CM* codes for hepatic encephalopathy are unreliable and there is no *ICD-10-CM* code, we used the first dispensed date for rifaximin or lactulose as a proxy.

Acute-on-chronic liver events were defined based on the model for end-stage liver disease (MELD) score. Among patients with cirrhosis, we calculated the following formula: MELD = 3.78 × log_bilirubin_ + 11.2 × log_international normalized ratio_ + 9.57 × log_creatinine_ + 6.43 with the constraint that laboratory values less than 1 were set equal to 1.^21^ This procedure resulted in a minimum MELD score of 6.43 (higher MELD scores indicate higher levels of severity). Among patients with a MELD score less than 15, we used a 5-point increase in MELD score as a proxy for an acute-on-chronic liver event associated with increased mortality and morbidity.^22^ To rule out acute spikes in the MELD score due to transient conditions, such as infection, we required the MELD score change to persist for at least 90 days. We also deemed the change in the MELD score to have persisted if there was a liver transplant or death within 90 days following the initial 5-point increase.

We identified HBV reactivations using 3 methods in patients with^23,24^: (1) a history of positive hepatitis B core antibody and negative hepatitis B surface antigen at the time of initiating DAA therapy who became hepatitis B surface antigen–positive within 180 days after receiving a DAA; (2) undetectable levels of HBV DNA at the time of initiating DAA therapy who had a numeric result within 180 days after receiving a DAA; and (3) a numeric hepatitis B surface antigen result at the time of initiating DAA therapy whose viral load increased by a factor of 10 within 180 days after receiving a DAA. We further required that the reactivations be clinically significant: bilirubin level at least 3 mg/dL (to convert to micromoles per liter, multiply by 17.104), aspartate aminotransferase level at least 400 U/L, or alanine aminotransferase level at least 500 U/L (to convert aspartate aminotransferase and alanine aminotransferase to microkatals per liter, multiply by 0.0167).^25^

### Covariates

We included several covariates in our analysis: demographics (age, sex, race, and ethnicity), year, body mass index, smoking status, history of use (skilled nursing, home health, emergency department, inpatient), laboratory results (MELD score and aspartate aminotransferase, alanine aminotransferase, hemoglobin A_1c_, and albumin levels), and a calculated aspartate aminotransferase level to platelet ratio index score.^26^ We defined comorbidities using algorithms of Quan et al.^27^

We examined patterns of missing data for laboratory tests. The proportion of patients missing laboratory tests ranged from 0.5% for alanine aminotransferase levels to 29.2% for hemoglobin A_1c_ levels. We found no significant differences between the DAA and non-DAA groups in the rates at which these tests were missing. Missing data for laboratory values were imputed with the mean value at baseline. Missing data for other variables were rare and addressed through categorical assignment (race, ethnicity, and smoking status) or mean imputation (body mass index).

### Statistical Analysis

We calculated unadjusted adverse event rates by counting the number of individuals who experienced the event and dividing by the total exposure time among all eligible individuals. For each event, the follow-up time for each person ended when 1 of the following occurred: adverse event of interest, death, loss of membership or follow-up, or 180 days after a DAA was dispensed, whichever came first. Separate models of adverse event rates were estimated for each of the 3 systems and for each outcome. Patients were excluded if they had the adverse event of interest before their index date.

Marginal structural models (MSMs) were used to adjust time-to-event analyses for patient characteristics that may affect outcomes, probability of treatment, and probability of censoring.^28^ The MSM is like the pooled logistic regression approach to survival analysis, but^29^ MSMs have the added feature that the probability of a patient receiving the treatment is modeled to make the treated and untreated populations more comparable. The MSM adjusts for time-dependent covariates and time-dependent exposures using inverse-of-probability-of-treatment weights. This adjustment may be thought of as the generalization of propensity score inverse-of-probability-of-treatment weights to repeated treatment decisions over time. The MSM adjusts for selection bias due to censoring by loss to follow-up using inverse-of-probability-of-censoring weights. Results using standard methods (eg, Cox proportional hazards regression model with time-varying covariates) may be biased.^29^

The MSM weights in the outcome models use the estimated probabilities of treatment and censoring from logistic regressions that include both static and time-dependent covariates. Time-varying covariates, such as laboratory values and diagnoses, were updated only until DAA dispensing to prevent biasing treatment outcomes. The approach is an intent-to-treat model with patients staying in the treatment arm until the end of follow-up or censoring. The MSM outcome models include a subset of covariates used in the probability of treatment model (baseline values of age, MELD score, and cirrhosis) to gain some additional robustness from case-mix adjustment while avoiding numeric instability from low adverse event rates. A more detailed description of the method is in the eMethods in the Supplement. To assess the balance produced by the weights, we calculated weighted means for each covariate across untreated points and compared with the weighted mean across treated points (eTable 2 in the Supplement). We also assessed rates of hospitalization and emergency department visits as a more sensitive indicator of potential serious adverse events using Poisson regression with time-varying covariates.

All analyses were stratified by health system. Means and 95% CIs for rate ratios (RRs) were exponentiated from normal approximation intervals in the logarithmic scale. Similarly, logit scale coefficient estimates and their 95% CIs from the MSMs were exponentiated to the odds scale. Estimates from the 3 health systems were combined using random-effects modeling—a common combination technique in meta-analyses.^30^ In addition to combined estimates and their SEs, the method provided a heterogeneity estimate and test to inform the comparability of the estimates across systems. Tests of homogeneity were conducted at the 5% level.

We assessed the sensitivity of our results to the method of imputing missing laboratory data. The MSM method uses a simple, mean-based imputation method. We tested whether more advanced methods would affect the results. We assumed multivariate normality for the data and used the expectation-maximization algorithm to estimate the parameters and the Monte Carlo Markov chain method to impute. Several of the variables were skewed; therefore, we log-transformed variables before imputing and then back-transformed to the original scale. Because our results were not sensitive to imputation methods, we used the standard MSM approach. Findings were considered significant at *P* < .05, with 2-tailed testing. Analyses were conducted with SAS, version 9.4 (SAS Institute Inc) and Harvard MSM, macro version 2.24.2015.

## Results

As shown in Figure 1, 82 419 patients were eligible for the study and 33 808 met all inclusion criteria. Requiring a quantitative HCV RNA result or genotype had the largest association with eligibility (33 622 [41% reduction]). Table 1 displays patient characteristics by health system and treatment status at study entry. The mean (SD) age was 57.2 (10.6) years (range, 51.7 [12.6] to 58.4 [9.0]) years. Participants were more likely to be men (20 899 [61.8%]; range, 55.2%-64.5%), white (18 562 [54.9%]; range, 49.7%-64.8%), non-Hispanic (27 367 [80.9%]; range, 70.5%-97.4%), and overweight (mean [SD] body mass index, 28.2 (66.6); range, 27.0-28.6 [calculated as weight in kilograms divided by height in meters squared]). Few patients had been previously diagnosed with liver cancer (862 [2.5%]; range, 1.7%-7.3%) or cirrhosis (5313 [15.7%]; range, 13.4%-34.0%) or had received a liver transplant (661 [2.0%]; range, 1.3%-5.7%); 10 952 (32.4%; range, 28.1%-40.9%) had 3 or more comorbid conditions. The proportion of patients dispensed DAAs during the study varied (7796 of 15 074 [51.7%] in health system 1, 6649 of 13 932 [47.7%] in health system 2, and 1079 of 4802 [22.5%] in health system 3). These percentages are within the health systems—not the overall distribution of participants by system. Total person-years of exposure were 7207.2 in the DAA group and 64 823.5 in the non-DAA group.

**Table 1.** Characteristics of Study Population at Study Entry by Health System and Exposure Group

Characteristic	No. (%)					
	Health System 1		Health System 2		Health System 3	
	DAA	No DAA	DAA	No DAA	DAA	No DAA
No. of participants contributing ≥1 d to exposure time	7796	7278	6649	7283	1079	3723
Age, y						
Mean (SD)	58.4 (9.0)	58.0 (10.6)	57.8 (9.9)	57.2 (11.9)	55.8 (10.1)	51.7 (12.6)
18-44	553 (7.1)	698 (9.6)	631 (9.5)	957 (13.1)	139 (12.2)	1016 (25.9)
45-64	5622 (72.1)	5000 (68.7)	4612 (69.4)	4625 (63.5)	841 (74.1)	2528 (64.3)
65-88	1621 (20.8)	1580 (21.7)	1406 (21.1)	1701 (23.4)	155 (13.7)	386 (9.8)
Sex						
Men	4714 (60.5)	4551 (62.5)	4122 (62.0)	4694 (64.5)	626 (55.2)	2192 (55.8)
Women	3082 (39.5)	2727 (37.5)	2527 (38.0)	2589 (35.5)	509 (44.8)	1738 (44.2)
Race						
American Indian or Alaska Native	59 (0.8)	80 (1.1)	51 (0.8)	32 (0.4)	NR^a^	NR^a^
Asian or Pacific Islander	552 (7.1)	454 (6.2)	376 (5.7)	349 (4.8)	12 (1.1)	20 (0.5)
Black	1147 (14.7)	1365 (18.8)	1225 (18.4)	1283 (17.6)	327 (30.3)	1190 (32.0)
White	4527 (58.1)	3864 (53.1)	3449 (51.9)	3617 (49.7)	694 (64.3)	2411 (64.8)
Other	490 (6.3)	433 (5.9)	173 (2.6)	202 (2.8)	37 (3.4)	85 (2.3)
Unknown	1021 (13.1)	1082 (14.9)	1375 (20.7)	1800 (24.7)	NR^a^	13 (0.3)
Hispanic ethnicity	1169 (15.0)	1236 (17.0)	1738 (26.1)	2146 (29.5)	51 (4.4)	101 (2.6)
BMI, mean (SD)	28.4 (5.7)	28.2 (5.9)	28.6 (5.6)	28.3 (5.9)	28.4 (6.4)	27.0 (6.2)
History of smoking	4782 (61.3)	4677 (64.3)	4343 (65.3)	5060 (69.5)	764 (70.8)	2833 (76.1)
Liver-related diagnoses at study entry						
Liver cancer	136 (1.7)	196 (2.7)	139 (2.1)	166 (2.3)	79 (7.3)	146 (3.9)
Liver transplant	117 (1.5)	95 (1.3)	191 (2.9)	109 (1.5)	62 (5.7)	87 (2.3)
Cirrhosis	1042 (13.4)	995 (13.7)	1079 (16.2)	1117 (15.3)	367 (34.0)	713 (19.2)
Decompensated cirrhosis, % of cirrhotic	339 (32.5)	435 (43.7)	377 (34.9)	446 (39.9)	139 (37.9)	366 (51.3)
Ascites, % of cirrhotic	221 (21.2)	313 (31.5)	245 (22.7)	310 (27.8)	102 (27.8)	268 (37.6)
Hemorrhagic varices, % of cirrhotic	78 (7.5)	92 (9.2)	114 (10.6)	112 (10.0)	20 (5.4)	62 (8.7)
Encephalopathy drug dispensed (lactulose, rifaximin), % of cirrhotic	224 (21.5)	276 (27.7)	213 (19.7)	263 (23.5)	111 (30.2)	264 (37.0)
Laboratory values at study entry						
MELD score in patients with cirrhosis^b^						
<10	743 (71.3)	539 (54.2)	676 (62.7)	555 (49.7)	237 (64.6)	399 (56.0)
10-15	241 (23.1)	284 (28.5)	309 (28.6)	345 (30.9)	86 (23.4)	191 (26.8)
>15	58 (5.6)	172 (17.3)	94 (8.7)	217 (19.4)	44 (12)	123 (17.3)
Albumin <3.5 g/dL	479 (7.3)	909 (15.7)	912 (17.3)	1401 (25.2)	162 (15.9)	906 (26.2)
Platelets <90 000 × 10^3^/μL	468 (6.1)	521 (7.3)	513 (7.8)	673 (9.4)	126 (12.3)	340 (9.6)
Charlson Comorbidity Index						
Charlson score, mean (SD)	2.8 (2.7)	3.2 (3.2)	2.6 (2.6)	2.9 (2.9)	3.7 (3.2)	3.4 (3.4)
No. of comorbidities						
0	760 (9.7)	742 (10.2)	684 (10.3)	771 (10.6)	71 (6.6)	566 (15.2)
1	2562 (32.9)	2194 (30.1)	2479 (37.3)	2608 (35.8)	296 (27.4)	997 (26.8)
2	2109 (27.1)	1735 (23.8)	1617 (24.3)	1635 (22.4)	271 (25.1)	759 (20.4)
≥3	2365 (30.3)	2607 (35.8)	1869 (28.1)	2269 (31.2)	441 (40.9)	1401 (37.6)

Abbreviations: BMI, body mass index (calculated as weight in kilograms divided by height in meters squared); DAA, direct-acting antiviral; MELD, model for end-stage liver disease; NR, not reported.

SI conversion factors: To convert albumin to grams per liter, multiply by 10; platelets to ×10^9^ per liter, multiply by 1.

^a^ Counts 10 or lower.

^b^ Higher MELD scores indicate higher levels of severity.

The unadjusted rate of observed events per 1000 persons per year during exposed and unexposed time is reported in Table 2. Being in the DAA group was associated with significantly lower death rates (10.7 vs 33.7 events per 1000 person-years; RR, 0.32; 95% CI, 0.25-0.40). Seven of the other adverse clinical event RRs were significant and below 70%, favoring the DAA group: multiple organ failure (RR, 0.56; 95% CI, 0.44-0.72), liver cancer (RR, 0.62; 95% CI, 0.48-0.80), hepatic decompensation (RR, 0.62; 95% CI, 0.52-0.73), acute-on-chronic liver event (RR, 0.68; 95% CI, 0.56-0.84), AMI (RR, 0.64; 95% CI, 0.42-0.97), ischemic stroke (RR, 0.63; 95% CI, 0.42-0.95), and hemorrhagic stroke (RR, 0.47; 95% CI, 0.25-0.89). Being in the DAA group was associated with significantly lower rates of hospitalization (RR, 0.50; 95% CI, 0.48-0.52) and emergency department visits (RR, 0.65; 95% CI, 0.63-0.66). None of the unadjusted comparisons favored the non-DAA group. The size of the eligible population for HBV reactivation varied by method from 2308 for the first method (most sensitive) to 54 for the third method (most specific) and we observed only 1 clinically significant event.

**Table 2.** Comparison of Unadjusted Adverse Events per 1000 Person-Years

Event	No.^a^	Health System 1		No.^a^	Health System 2		No.^a^	Health System 3		No.^a^	Combined		Rate Ratio (95% CI)
		Events, No.	Rate/1000 PY		Events, No.	Rate/1000 PY		Events, No.	Rate/1000 PY		Events, No.	Rate/1000 PY (95% CI)	
Death													0.32 (0.25-0.40)
DAA	7796	32	8.8	6649	32	10.2	1079	13	28.3	15 524	77	10.7 (8.3-13.1)	
No DAA^b^	15 074	750	26.6	13 932	1033	37.3	4802	403	44.9	33 808	2186	33.7 (32.3-35.1)	
Multiple organ failure													0.56 (0.44-0.72)
DAA	7604	33	9.4	6491	28	9.2	1012	0^c^	15.9	15 107	68	9.7 (7.4-12.0)	
No DAA	14 673	428	15.7	13 611	465	17.3	4586	185	21.8	32 870	1078	17.2 (16.2-18.3)	
Liver cancer													0.62 (0.48-0.80)
DAA	7552	30	8.6	6404	23	7.6	949	11	26.8	14 905	64	9.3 (7.0-11.5)	
No DAA	14 726	380	13.9	13 596	423	15.8	4572	128	15.2	32 894	931	14.9 (13.9-15.8)	
Hepatic decompensation													0.62 (0.52-0.73)
DAA	6923	67	20.9	5786	60	22.1	787	22	65.3	13 496	149	23.8 (20-27.7)	
No DAA	13 543	797	32.3	12 518	921	38.1	3900	430	63.4	29 961	2148	38.6 (37-40.2)	
Acute-on-chronic liver event													0.68 (0.56-0.84)
DAA	7023	48	14.8	5606	44	16.7	692	10	33.8	13 321	102	16.5 (13.3-19.7)	
No DAA	12 861	474	21.0	11 373	551	25.0	2803	169	35.1	27 037	1194	24.1 (22.8-25.5)	
Acute myocardial infarction													0.64 (0.42-0.97)
DAA	7510	12	3.4	6438	11	3.6	1011	NR^c^	0.0	14 959	23	3.3 (2-4.7)	
No DAA	14 433	125	4.7	13 430	136	5.1	4517	61	7.2	32 380	322	5.2 (4.6-5.8)	
Ischemic stroke													0.63 (0.42-0.95)
DAA	7385	16	4.7	6396	NR^c^	2.0	977	NR^c^	7.0	14 758	25	3.6 (2.2-5.1)	
No DAA	14 166	183	6.9	13 366	104	3.9	4368	64	7.9	31 900	351	5.8 (5.1-6.4)	
Hemorrhagic stroke													0.47 (0.25-0.89)
DAA	7406	NR^c^	1.5	6414	NR^c^	1.7	982	0	0.0	14 802	10	1.5 (0.6-2.3)	
No DAA	14 166	64	2.4	13 366	78	2.9	4368	47	5.8	31 900	190	3.1 (2.6-3.5)	
Arrhythmia													0.68 (0.43-1.07)
DAA	6757	11	3.5	5865	NR^c^	1.8	774	NR^c^	11.4	13 396	19	3.2 (1.8-4.5)	
No DAA	12 999	96	3.9	12 302	116	4.7	3367	47	7.1	28 668	230	4.7 (4.1-5.2)	
Acute kidney failure													0.72 (0.61-0.84)
DAA	7304	73	21.6	6123	64	22.3	894	24	62.3	14 321	161	24.2 (20.5-28.0)	
No DAA	14 134	702	27.3	13 002	866	34.3	4111	397	54.4	31 247	1965	33.7 (32.2-35.2)	
Cancer (nonliver)													0.78 (0.65-0.94)
DAA	6575	53	17.4	5790	59	21.7	889	15	39.1	13 254	127	20.7 (17.1-24.3)	
No DAA	13 156	614	25.6	12 522	621	25.8	4265	235	30.6	29 943	1470	26.4 (25.0-27.7)	
Hospitalization													0.50 (0.48-0.52)
DAA	7796	1974	156.9	6649	1789	148.3	1079	496	324.5	15 524	4259	162.8 (157.9-167.7)	
No DAA^b^	15 074	7114	252.2	13 932	7938	286.7	4802	6032	672.0	33 808	21 084	325 (320.6-329.4)	
ED visit													0.65 (0.63-0.66)
DAA	7796	7136	567.4	6649	6381	529.1	1079	907	593.5	15 524	14 424	551.2 (542.3-560.2)	
No DAA^b^	15 074	23 378	828.8	13 932	21 734	785.0	4802	10 243	1141.2	33 808	55 355	853.4 (846.2-860.5)	

Abbreviations: DAA, direct-acting antiviral; ED, emergency department; NR, not reported; PY, person-years.

^a^ Number of participants who contributed 1 or more days of exposure to the rate calculations. Participants who were dispensed a DAA contributed exposure time to the no DAA rate calculation before being dispensed a DAA.

^b^ These counts include 55 patients whose no DAA exposure time ended on the same day they became eligible (18 in site 1, 32 in site 2, and 5 in site 3). Twenty-seven of these patients received a new DAA on the day they became eligible and started contributing DAA exposure time, 25 were censored by receiving an older DAA, and 3 were censored by end of membership. These cases contribute no exposure time and no events to the no DAA rate calculation.

^c^ Counts 10 or lower.

**Figure 2.**
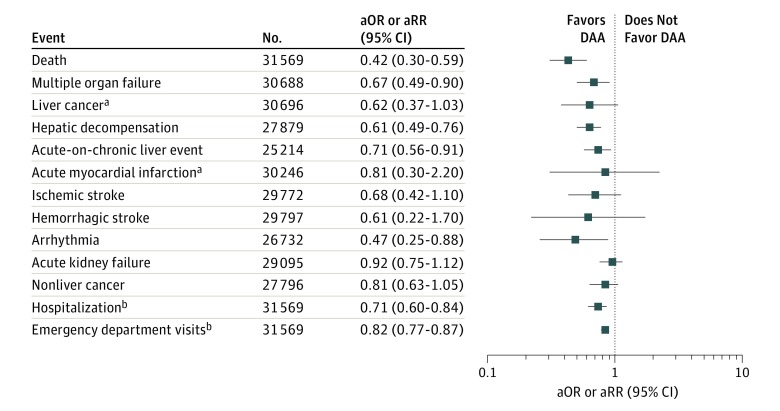
Adjusted Odds of Experiencing Adverse Events Among Those Exposed and Not Exposed to Direct-Acting Antiviral (DAA) Medications aOR indicates adjusted odds ratio; aRR, adjusted rate ratio. ^a^Failed test of homogeneity (heterogeneity estimate, 69% for liver cancer and 74% for acute myocardial infarction). ^b^Calculated as aRRs.

Figure 2 displays the MSM-adjusted combined estimated odds of experiencing an adverse event for patients who received a DAA compared with those who did not receive a DAA. The DAA exposures were associated with statistically significant lower odds of adverse events than non-DAA exposures for death (adjusted odds ratio [aOR], 0.42; 95% CI, 0.30-0.59), multiple organ failure (aOR, 0.67, 95% CI, 0.49-0.90), hepatic decompensation (aOR, 0.61; 95% CI, 0.49-0.76), acute-on-chronic liver event (aOR, 0.71; 95% CI, 0.56-0.91), and arrhythmia (aOR, 0.47; 95% CI, 0.25-0.88). We also observed significantly lower adjusted rates of hospitalizations (adjusted rate ratio [aRR], 0.71; 95% CI, 0.60-0.84) and emergency department visits (aRR, 0.82; 95% CI, 0.77-0.87).

For 2 of the adverse events (liver cancer and AMI), the test for homogeneity indicated significant heterogeneity in results by health system (eTable 3 in the Supplement). The adjusted odds of being diagnosed with liver cancer were significantly lower among those dispensed DAAs in health system 1 (aOR, 0.51; 95% CI, 0.32-0.80) and health system 2 (aOR, 0.44; 95% CI, 0.26-0.74). The aOR in health system 3 was not statistically significant but favored those not dispensed DAAs (aOR, 1.45; 95% CI, 0.65-3.22). No AMIs were observed in health system 3 among those dispensed DAAs; the results in health system 1 favored those not dispensed DAAs but were not significant (aOR, 1.57; 95% CI, 0.84-2.95); the odds of having an AMI in health system 2 were statistically significantly lower among those dispensed DAAs (aOR, 0.41; 95% CI, 0.20-0.83).

## Discussion

In this large cohort study conducted in 3 health systems, we found no evidence that DAA exposure was associated with a higher rate of serious adverse events. We examined multiple outcomes, including those related to liver, kidney, and cardiovascular systems, as well as hospitalizations and ED visits, and used rigorous statistical methods to address some of the threats to validity that are common in cohort studies.

This research contributes to the literature on the safety profile of this newer class of medications for treating HCV. No medication is without risks and multiple randomized trials have demonstrated the potential benefits associated with these agents.^9^ When patients and physicians are considering treatment options for HCV, they must determine whether the potential risks associated with the treatment outweigh the potential benefits. Because clinical trials are commonly conducted with participants who have different demographic and health profiles than patients who are subsequently offered medication therapy, the results of postmarketing studies, such as this one, that are based on real-world patients and their experiences can contribute a richer source of information for shared decision making.^31^ Our study included a higher proportion of patients from racial and ethnic minorities than in most of the trials to date (14.7%-32.0% black, 0.5%-7.1% Asian or Pacific Islander, 2.6%-29.5% Hispanic). Our study also included patients who are typically excluded from clinical trials, such as those with a previous diagnosis of liver cancer, prior liver transplant, cirrhosis, and multiple comorbidities.

Examining the safety of DAAs presents challenges because some of the outcomes of interest are also known complications of HCV. The underlying disease process can take more than 20 years to progress to clinically significant symptoms and most people with HCV do not develop these complications. It is challenging, particularly for liver-related outcomes, to determine whether the adverse events observed were caused by the medication or were part of the course of the disease. The comparison group that we constructed and our analytic methods are tools to parse these competing explanations in cohort studies. Our findings on liver cancer are consistent with other studies,^32^ including a recent cohort study in the Veterans Affairs system^33^ and a meta-analysis.^34^ Other adverse events, such as cardiovascular and kidney-related events, are more likely to be due to the drug than to the underlying disease. The findings related to emergency department visits and hospitalizations, which were included as a more sensitive indicator of potential adverse events, also favored the DAA group. Because we observed a consistent pattern across different types of adverse events, we have greater confidence that DAAs may not be associated with increased risks of serious adverse events.

The US Preventive Services Task Force^35^ recommends that persons who are at high risk for acquiring HCV (eg, past or current injection drug use, blood transfusion before 1992, significant direct percutaneous exposures) and those born between 1945 and 1965 be screened for HCV infection. Those who have HCV may consider, in consultation with their health care professional, whether treatment is appropriate. For patients who are otherwise apparently healthy, the decision to use a medication that could cause a health problem can be particularly difficult. The adverse event profile that we observed herein should contribute useful information for those who face this decision. For patients who are already experiencing significant health effects of HCV, this study may provide evidence that DAAs are not associated with higher adverse event rates.

### Strengths and Limitations

The consistency in results across 3 large, demographically diverse health systems in 2 different regions of the country is a strength of the study and provides a greater measure of confidence in the conclusions than a single-site study. The health systems have comprehensive clinical data available, which enabled us to control for a variety of demographic and clinical characteristics using rigorous statistical methods. The ability to reasonably rapidly address questions of importance to patients demonstrates the potential for ongoing and robust monitoring of drug safety using real-world data, particularly when the events are rare or might be triggered by other factors, such as comorbidities or other medications, that typically lead to patients being excluded from trials.

Our study also has some limitations. This was a cohort study and is subject to the known biases for such designs. Although we had a rich collection of clinical data available, it is likely that some of our results may be explained by unmeasured confounding by indication. Although there were no formal restrictions on access to treatment in the systems, it is likely that the decision to treat was different in the earliest days of DAA availability. We controlled for year and model the decision to treat to address this possible factor. In this study, confounding by indication or selection bias was complicated by the possibility of competing confounding mechanisms. Our findings are consistent with a bias toward healthier patients receiving DAAs (eg, lower proportion with 2 or more comorbidities among those dispensed DAAs). However, some of the associations are complex. For example, we observed that people with cirrhosis were more likely to be treated with DAAs (consistent with a bias toward sicker patients) but, among those with cirrhosis, MELD scores were lower among the treated patients (consistent with a bias toward healthier individuals). Differences that we observed between the health systems might be explained by different channeling mechanisms at work in each system.

## Conclusions

We found no evidence that patients dispensed DAAs experienced higher rates of adverse liver-, cardiovascular-, kidney-, or emergency department visit and hospitalization–related events in 3 health systems. Although it is tempting to conclude that DAAs are protective against many serious adverse outcomes, these outcomes may be a consequence of channeling healthier patients to DAA treatment. A more conservative conclusion is that DAA exposure may not be associated with higher rates of adverse events.

## Notes

Supplement. **eTable 1.** Definitions of Outcome and Covariate Variables Used in the Study **eMethods.** Additional Methodologic Details on the Marginal Structural Modeling Method **eTable 2.** Differences Between Treated and Untreated Before and After Applying Marginal Structural Modeling Weights **eTable 3.** Marginal Structural Modeling Adjusted Odds Ratio (OR) or Rate Ratio (RR) of Adverse Events by Health System and Combined
