# Supplementary material for: Assessing the Safety of Direct-Acting Antiviral Agents for Hepatitis C
Source: JAMA Netw Open. 2019 Jun 7;2(6):e194765. doi: 10.1001/jamanetworkopen.2019.4765 (PMC6563580; doi:10.1001/jamanetworkopen.2019.4765)
Supplement: Supplement. — eTable 1. Definitions of Outcome and Covariate Variables Used in the Study eMethods. Additional Methodologic Details on the Marginal Structural Modeling Method eTable 2. Differences Between Treated and Untreated Before and After Applying Marginal Structural Modeling Weights eTable 3. Marginal Structural Modeling Adjusted Odds Ratio (OR) or Rate Ratio (RR) of Adverse Events by Health System and Combined [file jamanetwopen-2-e194765-s001.pdf]

Supplementary Online Content

McGlynn EA, Adams JL, Kramer J, et al. Assessing the safety of direct-acting antiviral agents for hepatitis C. *JAMA Netw Open*. 2019;2(6):e194765. doi:10.1001/jamanetworkopen.2019.4765

eTable 1. Definitions of Outcome and Covariate Variables Used in the Study

eMethods. Additional Methodologic Details on the Marginal Structural Modeling Method

eTable 2. Differences Between Treated and Untreated Before and After Applying Marginal Structural Modeling Weights

eTable 3. Marginal Structural Modeling Adjusted Odds Ratio (OR) or Rate Ratio (RR) of Adverse Events by Health System and Combined

This supplementary material has been provided by the authors to give readers additional information about their work.

© 2019 McGlynn EA et al. *JAMA Network Open.*

eTable 1. Definitions of Outcome and Covariate Variables Used in the Study

Diagnoses	ICD Codes or Definition	Data Source
Acute Kidney Failure	**ICD-9** 584.x<br>**ICD-10** N17.x	Inpatient or Outpatient
Acute Myocardial Infarction	**ICD-9** 410.x<br>**ICD-10** I21.x	Inpatient
Acute on chronic liver event	A patient will be characterized as having an acute on chronic liver event if they have an acute change in MELD score of 5 or more within a 6-month period.<br><br>Eligibility: MELD < 15 at baseline<br><br>The change is deemed to have persisted (defined as meeting one of the following criteria: MELD continues to be elevated 3 months later, liver transplant, death).	Inpatient or Outpatient labs
Alcohol Abuse	**ICD-9** 265.2, 291.1–291.3, 291.5–291.9, 303.0, 303.9, 305.0, 357.5, 425.5, 535.3, 571.0–571.3, 980.x, V11.3<br>**ICD-10** F10.x, E52, G62.1, I42.6, K29.2, K70.0, K70.3, K70.9, T51.x, Z50.2, Z71.4, Z72.1	Inpatient or Outpatient
Arrhythmia	**ICD-9** 427.1, 427.42, 427.5, 427.9<br>**ICD-10** I47.2, I49.01, I49.02, I46.9, I49.9	Inpatient
Ascites	**ICD-9** 789.5, 789.51, 789.59<br>**ICD-10** R18.0, R18.8, K71.51, K70.11, K70.31	Inpatient or Outpatient
Cerebrovascular Disease[26]	**ICD-9** 362.34, 430.x–438.x<br>**ICD-10** G45.x, G46.x, H34.0, I60.x–I69.x	Inpatient or Outpatient
Change in eGFR	20% decline in eGFR within 1 year	
Change in HbA1c	A change of 1 percentage point or more within 1 year	Inpatient or Outpatient Labs
Chronic Pulmonary Disease[26]	**ICD-9** 416.8, 416.9, 490.x–505.x, 506.4, 508.1, 508.8<br>**ICD-10** I27.8, I27.9, J40.x–J47.x, J60.x–J67.x, J68.4, J70.1, J70.3	Inpatient or Outpatient
Cirrhosis	**ICD-9** 571.5, 571.6,<br>**ICD-10** K74.0, K74.3, K74.5, K74.5, K74.60, K74.69	Inpatient or Outpatient
Congestive Heart Failure[26]	**ICD-9** 398.91, 402.01, 402.11, 402.91, 404.01, 404.03, 404.11, 404.13, 404.91, 404.93, 425.4–425.9, 428.x<br>**ICD-10** I09.9, I11.0, I13.0, I13.2, I25.5, I42.0, I42.5–I42.9, I43.x, I50.x, P29.0	Inpatient or Outpatient

© 2019 McGlynn EA et al. *JAMA Network Open.*

Death	Indicator that patient had died	Site 1: EHR, membership, and Social Security Administration<br>Site 2: EHR, membership, and Social Security Administration<br>Site 3: EHR, membership, and Social Security Administration
Decompensation	Composite variable that counts the first time any of the following events occur: jaundice, ascites, variceal hemorrhage, hepatic encephalopathy	Inpatient or Outpatient
Dementia[26]	**ICD-9** 290.x, 294.1, 331.2<br>**ICD-10** F00.x–F03.x, F05.1, G30.x, G31.1	Inpatient or Outpatient
Depression	**ICD-9** 296.2, 296.3, 296.5, 300.4, 309.x, 311<br>**ICD-10** F20.4, F31.3–F31.5, F32.x, F33.x, F34.1, F41.2, F43.2	Inpatient or Outpatient
Diabetes with Chronic Complications[26]	**ICD-9** 250.4–250.7<br>**ICD-10** E10.2–E10.5, E10.7, E11.2–E11.5, E11.7, E12.2-E12.5, E12.7, E13.2–E13.5, E13.7, E14.2–E14.5, E14.7	Inpatient or Outpatient
Diabetes without Chronic Complications[26]	**ICD-9** 250.0–250.3, 250.8, 250.9<br>**ICD-10** E10.0, E10.1, E10.6, E10.8, E10.9, E11.0, E11.1, E11.6, E11.8, E11.9, E12.0, E12.1, E12.6, E12.8, E12.9, E13.0, E13.1, E13.6, E13.8, E13.9, E14.0, E14.1, E14.6, E14.8, E14.9	Inpatient or Outpatient
Drug Abuse	**ICD-9** 292.x, 304.x, 305.2–305.9, V65.42<br>**ICD-10** F11.x–F16.x, F18.x, F19.x, Z71.5, Z72.2	Inpatient or Outpatient
HBV reactivation	A patient will be characterized as having a clinically significant HBV reactivation if they have any of the following indications:<br>AST > 400 U/L<br>ALT > 500 U/L<br>INR > 1.3<br>Bilirubin > 3	Outpatient labs
HCV	**ICD-9** 070.44, 070.54, 070.70, 070.71, V02.62<br>**ICD-10** B18.2, B19.20, B19.21, Z22.52	Inpatient or Outpatient
Hemiplegia or Paraplegia[26]	**ICD-9** 334.1, 342.x, 343.x, 344.0–344.6, 344.9<br>**ICD-10** G04.1, G11.4, G80.1, G80.2, G81.x, G82.x, G83.0–G83.4, G83.9	Inpatient or Outpatient
Hemorrhagic Stroke	**ICD-9** 430.x, 431.x, 432.x<br>**ICD-10** I60.x, I61.x, I62.x	Inpatient
Hepatic Encephalopathy	Date of outcome is first prescription for lactulose, rifaximin, or xifaxan	Inpatient or Outpatient Dispense

© 2019 McGlynn EA et al. *JAMA Network Open.*

Hepatorenal Syndrome	**ICD-9** 572.4<br>**ICD-10** K76.7	Inpatient or Outpatient
HIV/AIDS	**ICD-9** V08, 042, 043, 044<br>**ICD-10** Z21, B20	Inpatient or Outpatient
Hyperlipidemia	**ICD-9** 272.0, 272.2, 272.4<br>**ICD-10** E78.0, E78.2, E78.4, E78.5	Inpatient or Outpatient
Hypertension	**ICD-9** 401.x, 402.x, 403.x, 404.x, 405.x<br>**ICD-10** I10.x, I11.x, I12.x, I13.x, I15.x	Inpatient or Outpatient
Hyponatremia	**ICD-9** 276.1<br>**ICD-10** E87.1	Inpatient or Outpatient
Ischemic Stroke	**ICD-9** 433.x, 434.x<br>**ICD-10** I63.x, I65.x	Inpatient
Jaundice	**ICD-9** 782.4<br>**ICD-10** R17	Inpatient or Outpatient
Liver Cancer	**ICD-9** 155.x<br>**ICD-10** C22.x	Inpatient or Outpatient
Liver Transplant	**ICD-9** V42.7<br>**ICD-10** Z94.4	Inpatient
Malignancy, including lymphoma, leukemia, excluding malignant neoplasm of skin[26]	**ICD-9** 140.x–172.x, 174.x–195.8, 200.x–208.x, 238.6<br>**ICD-10** C00.x–C26.x, C30.x–C34.x, C37.x–C41.x, C43.x, C45.x–C58.x, C60.x–C76.x, C81.x–C85.x, C88.x, C90.x–C97.x	Inpatient or Outpatient
Metastatic Solid Tumor[26]	**ICD-9** 196.x–199.x<br>**ICD-10** C77.x–C80.x	Inpatient or Outpatient
Mild Liver Disease[26]	**ICD-9** 070.22, 070.23, 070.32, 070.33, 070.44, 070.54, 070.6, 070.9, 570.x, 571.x, 573.3, 573.4, 573.8, 573.9, V42.7<br>**ICD-10** B18.x, K70.0–K70.3, K70.9, K71.3–K71.5, K71.7, K73.x, K74.x, K76.0, K76.2–K76.4, K76.8, K76.9, Z94.4	Inpatient or Outpatient
Moderate or Severe Liver Disease[26]	**ICD-9** 456.0–456.2, 572.2–572.8<br>**ICD-10** I85.0, I85.9, I86.4, I98.2, K70.4, K71.1, K72.1, K72.9, K76.5, K76.6, K76.7	Inpatient or Outpatient
Multiple Organ Failure	**ICD-9** 995.92, 995.94, 785.52<br>**ICD-10** R65.11, R65.2x	Inpatient
Myocardial Infarction, History	**ICD-9** 412.x(old mi)<br>**ICD-10** I22.x, I25.2	Inpatient or Outpatient
Other Cancers	**ICD-9** 140.x – 208.x (except 155.x)<br>**ICD-10** C00-C97 (except C22.x)	Inpatient
Peptic Ulcer Disease[26]	**ICD-9** 531.x–534.x<br>**ICD-10** K25.x–K28.x	Inpatient or Outpatient

© 2019 McGlynn EA et al. *JAMA Network Open.*

Peripheral Vascular Disease[26]	**ICD-9** 093.0, 437.3, 440.x, 441.x, 443.1–443.9, 47.1, 557.1, 557.9, V43.4<br>**ICD-10** I70.x, I71.x, I73.1, I73.8, I73.9, I77.1, I79.0, I79.2, K55.1, K55.8, K55.9, Z95.8, Z95.9	Inpatient or Outpatient
Portal Hypertension	**ICD-9** 572.3<br>**ICD-10** K76.6	Inpatient or Outpatient
Psychoses	**ICD-9** 293.8, 295.x, 296.04, 296.14, 296.44, 296.54, 297.x, 298.x<br>**ICD-10** F20.x, F22.x–F25.x, F28.x, F29.x, F30.2, F31.2, F31.5	Inpatient or Outpatient
Renal Disease[26]	**ICD-9** I12.0, I13.1, N03.2–N03.7, N05.2–N05.7, N18.x, N19.x, N25.0, Z49.0–Z49.2, Z94.0, Z99.2<br>**ICD-10** 403.01, 403.11, 403.91, 404.02, 404.03, 404.12, 404.13, 404.92, 404.93, 582.x, 583.0–583.7, 585.x, 586.x, 588.0, V42.0, V45.1, V56.x	Inpatient or Outpatient
Rheumatic Disease[26]	**ICD-9** 446.5, 710.0–710.4, 714.0–714.2, 714.8, 725.x<br>**ICD-10** M05.x, M06.x, M31.5, M32.x–M34.x, M35.1, M35.3, M36.0	Inpatient or Outpatient
Variceal Hemorrhage	**ICD-9** 456.0, 456.20<br>**ICD-10** I85.01, I85.11	Inpatient or Outpatient
Varices, Non-hemorrhagic	**ICD-9** 456.1, 456.21,<br>**ICD-10** I85.00, I85.10	Inpatient or Outpatient

Note: diagnostic variables in the MSM are time-varying binary variables that are a 1 if the patient has received the ICD code prior to each MSM time period

**Laboratory Tests**	**Description**
Bilirubin	LOINC code: 1968-7, 1975-2, 1971-1, 15448-1, 42719-5, 59827-6, 59828-4, 50551-1, 5770-3, 58450-8, 70199-5, 1978-6, 1977-8
INR	LOINC code: 6301-6, 34714-6, 4617-2
Creatinine	LOINC code: 2160-0
HBsAG	LOINC code: 10674-0, 10675-7, 5195-3, 5196-1, 5197-9, 65633-0, 709376, 7905-3
HBV DNA	LOINC code: 16934-2, 20442-0, 23869-1, 29615-2, 42595-9, 45161-7, 48398-2, 5007-0, 5008-8, 5009-6
HBV Antibody	HBcAB<br>LOINC code: 32685-0, 5185-4, 31204-1, 16933-4, 13952-7, 22316-4, 24113-3, 5187-0, 51914-0<br>HBeAB<br>LOINC code: 22320-6, 13953-5
ALT	LOINC code: 1742-6
AST	LOINC code: 1920-8
Platelets	LOINC code: 777-3, 26515-7
Albumin	LOINC code: 1751-7, 76631-1, 61151-7, 61152-5, 2862-1

© 2019 McGlynn EA et al. *JAMA Network Open.*

HbA1c	LOINC code: 17855-8, 4548-4
HCV Genotype	LOINC 32286-7
HCV Viral Load	LOINC code: 11011-4, 20416-4, 20571-6, 34703-9, 38180-6, 49605-9
HCV Antibody	LOINC code: 13955-0, 16128-1, 22327-1, 22329-7

Note: lab variables in the MSM are time-varying and are the most recently available lab prior to each MSM time period

**Direct Acting Antivirals**		
**Generic**	**Brand Name**	**NDC code**
Daclatasvir	Daklinza	00003001101
Daclatasvir	Daklinza	00003021301
Daclatasvir	Daklinza	00003021501
Elbasvir/Grazoprevir	Zepatier	00006307401
Elbasvir/Grazoprevir	Zepatier	00006307402
Ombitasvir/Paritaprevir/Ritonavir/Dasabuvir	Viekira	00074006328
Glecaprevir/Pibrentasvir	Mavyret	00074262528
Ombitasvir/Paritaprevir/Ritonavir	Technivie	00074308228
Ombitasvir/Paritaprevir/Ritonavir/Dasabuvir	Viekira	00074309328
Boceprevir	Victrelis	00085031402
Boceprevir	Victrelis	00085031402
Telaprevir	Incivek	51167010001
Telaprevir	Incivek	51167010003
Simeprevir	Olysio	59676022528
Sofosbuvir	Sovaldi	61958150101
Ledipasvir/Sofosbuvir	Harvoni	61958180101
Sofosbuvir/Velpatasvir	Epclusa	61958220101

© 2019 McGlynn EA et al. *JAMA Network Open.*

Utilization	Description
Emergency Department	At least 1 emergency department visit in the past year
Inpatient Hospitalization	At least 1 inpatient hospitalization in the past year
SNF	At least 1 visit to a skilled nursing facility in the past year
Home Health	At least 1 home health visit in the past year

Note: utilization variables in the MSM are time-varying and are updated prior to each MSM time period

© 2019 McGlynn EA et al. *JAMA Network Open.*

eMethods. Additional Methodologic Details on the Marginal Structural Modeling Method

Marginal Structural Modeling (MSM) can be thought of as the extension of propensity score weighting to treatment decisions over time. In propensity score modeling, the comparison and treatment arms are weighted to match the treatment of interest on baseline covariates. MSM extends this idea to modeling repeated opportunities to initiate treatment over time.

Following the notation of Robins et al. 2000, let V_i denote the vector of baseline static covariates, let L_{ik} denote the vector of time-varying covariates, and let A_{ik} denote the treatment group, and Y_{ik} denote the outcome for person i at time k.

We fit the pooled logistic regression model

$\text{logit}(P_{ik}) = \beta_0 + \beta_1 a_{ik} + \vec{\beta}_V \vec{V_i}$

where $P_{ik} = P(Y_{ik} = 1)$ and each observed outcome Y_{ik} was weighted using the product of stabilized inverse probability of treatment weights (IPTW) and stabilized inverse probability of censoring weights (ICPW).

For each time period k, we fit the following logistic regression model for the treatment received, conditional on all observed confounders.

$$logit\big(P(A_{ik} = 1 \mid A_{i(k-1)}, L_{ik}, V_i)\big) = \gamma_{0k} + \vec{\gamma}_L \vec{L}_{ik} + \vec{\gamma}_V \vec{V}_{ik}.$$

Note that we performed an intent-to-treat analysis, meaning once an individual received DAA, they were in the DAA treatment group for the remainder of the study, so

$P(A_{ik} = 1 \mid a_{i(k-1)} = 1, L_{ik}, V_i) = 1.$

Since inverse probability of treatment weight (IPTW)

$W_{ik} = \prod_{t=0}^{k} \frac{1}{P(A_{it} = a_{it} \mid a_{i(t-1)}, L_{it}, V_i)}$. tends to be highly variable due to small probabilities in the denominator leading to large weights, we used the stabilized weight

© 2019 McGlynn EA et al. *JAMA Network Open.*

$$SW_{ik} = \prod_{t=0}^{k} \frac{P(A_{it} = a_{it} \mid a_{i(t-1)})}{P(A_{it} = a_{it} \mid a_{i(t-1)}, L_{it}, V_i)}.$$

The stabilized weight preserves unbiased estimation of the causal effects of treatment while maintaining the same effective sample size as the original dataset. We repeated the above modeling for censoring and obtain stabilized weights of $SW_{ik} * SW_{ik}^{C}$. Weights above the 99th percentile or below the 1st percentile were truncated to the corresponding percentile.

Weighting each observation using IPTW effectively creates a pseudo-population of subjects in which treatment is no longer confounded by V or L, thus $\hat{\beta}_1$ is an unbiased estimator of the causal effect of treatment, if there is no unmeasured confounding.

© 2019 McGlynn EA et al. *JAMA Network Open.*

eTable 2. Differences Between Treated and Untreated Groups Before and After Applying Marginal Structural Modeling Weights

	Health System 1				Health System 2				Health System 3			
	Treated	Untreated	Treated_W	Untreated_W	Treated	Untreated	Treated_W	Untreated_W	Treated	Untreated	Treated_W	Untreated_W
A1c	6.0	6.2	6.1	6.2	6.1	6.2	6.1	6.2	6.1	6.1	6.1	6.1
Acute Kidney Failure	6.5	9.2	8.1	8.1	8.3	9.4	9.2	8.8	16.1	19.0	19.2	18.0
Acute Myocardial Infarction	4.0	5.4	4.8	4.8	3.6	4.5	4.0	4.1	6.4	7.6	7.7	7.2
Albumin	4.1	4.1	4.1	4.1	3.8	3.8	3.8	3.8	4.0	3.9	4.0	3.9
Alcohol Abuse	9.3	9.3	11.6	9.5	10.3	9.9	11.4	10.2	20.9	21.1	23.1	20.9
ALT	34.1	57.7	30.6	60.2	37.1	60.4	33.9	62.2	37.6	51.8	37.6	51.9
APRI	0.6	0.8	0.5	0.9	0.7	0.9	0.7	0.9	0.7	0.9	0.7	0.9
Arrhythmia	16.0	17.8	17.2	17.0	14.3	15.7	15.0	15.1	32.0	36.9	33.1	35.8
Ascites	5.7	5.9	7.5	6.0	6.2	5.6	6.5	6.0	16.5	12.9	15.6	12.9
AST	36.2	51.8	33.4	53.6	42.5	53.1	39.2	54.6	40.0	53.3	40.3	53.3
Cardiovascular Disease	8.0	10.4	9.3	9.4	6.0	7.1	6.1	6.5	11.8	15.4	13.7	14.3
Cirrhosis	25.4	15.1	22.6	18.9	28.5	19.2	25.0	22.7	53.5	28.6	42.3	31.7
Coronary Heart Failure	4.9	7.7	6.3	6.7	4.5	6.8	5.6	5.8	14.7	14.7	15.0	14.2
CPD	42.6	42.9	43.3	42.4	33.2	32.5	33.6	32.4	36.4	39.5	39.0	38.6
Dementia	0.4	1.5	0.8	1.1	0.5	1.7	1.1	1.2	1.8	2.4	2.3	2.2
Depression	44.4	44.5	45.2	44.3	34.5	34.9	35.7	34.6	43.9	46.4	47.1	46.0
Diabetes	21.3	23.7	22.7	22.7	24.8	25.7	24.5	25.2	34.4	28.9	32.8	28.7
Diabetes with complications	11.2	14.2	12.4	12.9	12.7	14.6	12.9	13.7	14.5	12.6	14.4	12.1
Drug Abuse	27.1	31.7	30.5	30.1	19.4	23.0	23.4	21.9	36.5	49.4	44.5	47.6
ED*	27.9	32.3	32.1	31.4	27.6	30.2	30.0	29.4	33.4	39.1	35.4	38.5
Hemorrhagic Stroke	1.1	1.4	1.2	1.2	0.8	1.2	1.0	1.0	1.2	2.7	2.3	2.4
HIV	3.5	3.2	3.7	3.4	2.4	2.2	2.3	2.3	7.6	8.0	7.8	7.8
Home Health*	2.7	4.2	3.0	3.9	4.5	6.7	5.3	6.0	-**	-**	-**	-**

© 2019 McGlynn EA et al. *JAMA Network Open.*

Hyperlipidemia	27.7	30.3	28.5	28.9	33.1	33.8	33.7	33.0	19.7	12.7	16.7	13.0
Hypertension	51.7	54.2	52.5	52.8	53.5	54.9	52.4	53.9	45.5	32.4	40.2	33.1
Hyponatremia	6.1	7.3	7.5	6.8	6.9	8.4	8.0	7.7	11.7	14.5	13.6	13.6
Inpatient*	9.0	12.1	11.0	11.6	10.1	13.3	11.4	12.6	20.5	29.8	24.8	28.8
Ischemic Stroke	5.9	7.6	6.9	6.9	4.1	4.8	4.1	4.4	8.4	11.2	10.1	10.5
Jaundice	1.5	1.6	1.9	1.6	1.5	1.4	1.7	1.5	3.8	3.8	4.2	3.6
Liver Cancer	3.4	3.0	4.6	3.3	3.9	3.3	4.4	3.6	10.6	6.0	8.7	6.3
Liver Transplant	2.4	1.6	2.6	1.9	3.9	2.5	3.6	3.1	7.3	4.2	6.6	4.4
Malignancy	12.5	12.8	13.5	12.6	11.4	12.0	12.3	11.7	19.7	16.4	20.0	16.3
MELD	7.8	8.1	8.1	7.9	8.1	8.3	8.3	8.2	8.8	9.1	9.0	9.0
Mild Liver Disease	97.0	90.6	93.0	91.8	95.7	93.4	94.0	93.7	99.5	83.6	98.7	85.2
Moderate or Severe Liver Disease	11.9	7.4	11.9	9.2	13.6	9.8	13.0	11.4	28.4	17.0	24.3	18.1
Multiple Organ Failure	2.7	3.8	3.3	3.4	2.7	3.3	3.0	3.0	4.1	5.7	4.7	5.3
Other Cancers	16.0	15.2	15.6	15.3	13.3	13.5	13.4	13.2	15.9	14.6	16.8	14.4
Peripheral Vascular Disease	26.5	27.0	28.2	26.0	20.8	22.7	21.2	21.4	22.3	22.6	23.7	21.7
Plegia	1.7	2.3	2.1	2.0	1.4	1.9	1.5	1.7	2.1	3.5	2.7	3.2
Portal Hypertension	7.9	5.3	8.7	6.5	10.4	7.5	10.0	8.7	24.0	13.0	20.2	14.1
Psych	4.1	6.1	5.3	5.3	3.6	4.5	4.2	4.1	7.7	12.2	10.1	11.6
Renal	10.8	14.4	12.5	12.9	12.9	15.5	13.5	14.2	21.9	20.5	22.8	19.8
Rheumatic Disease	3.9	4.0	4.0	3.9	3.6	3.4	3.3	3.4	7.0	5.9	6.8	6.0
Skilled Nursing*	0.7	1.7	1.2	1.5	0.7	1.4	0.8	1.2	**	-**	-**	-**
Tumor	2.2	2.7	2.5	2.5	1.4	2.2	1.7	1.9	3.6	3.9	4.1	3.6
Ulcer	4.8	4.9	4.9	4.8	4.0	4.5	4.2	4.3	7.8	7.1	7.2	6.9
Varices, hemorrhagic	1.4	1.3	1.8	1.4	2.3	1.9	2.4	2.1	2.7	2.4	3.2	2.4
Varices, non-hemorrhagic	7.9	4.6	7.7	5.9	9.5	6.4	8.9	7.7	17.1	8.6	13.2	9.5

© 2019 McGlynn EA et al. *JAMA Network Open.*

** =Data element not available
*=%with encounter type within past year
_w = weighted with MSM weights
Abbreviations: A1c: hemoglobin A1c; ALT: alanine aminotransferase; APRI: AST to Platelet Ration; AST: aspartate aminotransferase; CPD: chronic pulmonary disease; ED: emergency department; HIV: Human immunodeficiency virus; MELD: model for end stage liver disease.

© 2019 McGlynn EA et al. *JAMA Network Open.*

eTable 3. Marginal Structural Modeling Adjusted Odds Ratio (OR) or Rate Ratio (RR) of Adverse Events by Health System and Combined

	Health System 1		Health System 2		Health System 3		Meta-Analysis		
**Event**	**Odds Ratio or Rate Ratio**	**95% CI**	**Odds Ratio or Rate Ratio**	**95% CI**	**Odds Ratio or Rate Ratio**	**95% CI**	**P-value, Test of Homogeneity**	**Combined Odds Ratio or Rate Ratio**	**95% CI**
Death	0.45	(0.30-0.67)	0.31	(0.21-0.46)	0.66	(0.35-1.24)	0.11	0.42	(0.30-0.59)
Multiple Organ Failure	0.67	(0.41-1.09)	0.60	(0.39-0.92)	0.97	(0.43-2.16)	0.59	0.67	(0.49-0.90)
Liver Cancer	0.51	(0.32-0.80)	0.44	(0.26-0.74)	1.45	(0.65-3.22)	0.04*	0.62	(0.37-1.03)
Hepatic Decompensation	0.58	(0.44-0.78)	0.55	(0.39-0.77)	0.87	(0.53-1.43)	0.30	0.61	(0.49-0.76)
Acute-on-Chronic Liver Event	0.78	(0.55-1.14)	0.64	(0.44-0.91)	0.73	(0.29-1.85)	0.70	0.71	(0.56-0.91)
Acute Myocardial Infarction	1.57	(0.84-2.95)	0.41	(0.20-0.83)	**	**	0.02*	0.81	(0.30-2.20)
Ischemic Stroke	0.70	(0.40-1.23)	0.41	(0.10-1.68)	0.90	(0.26-3.07)	0.70	0.68	(0.42-1.10)
Hemorrhagic Stroke	0.92	(0.20-4.21)	0.42	(0.10-1.73)	**	**	0.76	0.61	(0.22-1.70)
Arrhythmia	0.64	(0.28-1.46)	0.33	0.14-0.80)	**	**	0.56	0.47	(0.25-0.88)
Acute Kidney Failure	0.93	(0.71-1.23)	0.78	(0.58-1.05)	1.21	(0.77-1.91)	0.27	0.92	(0.75-1.12)
Cancer (non-liver)	0.68	(0.50-0.94)	0.80	(0.58-1.10)	1.26	(0.71-2.22)	0.19	0.81	(0.63-1.05)
Hospitalization***	0.60	(0.57-0.63)	0.82	(0.78-0.86)	0.73	(0.67-0.81)	< 0.01*	0.71	(0.60-0.84)
Emergency Department Visits***	0.78	(0.75-0.80)	0.87	(0.85-0.89)	0.81	(0.76-0.87)	< 0.01*	0.82	(0.77-0.87)

*p-value ≤.05

**Zero events observed in 180 days after receiving DAA

***Analyses performed using Poisson regression, estimates are rate ratios rather than odds ratios

© 2019 McGlynn EA et al. *JAMA Network Open.*
